# The association between GPS‐derived external workload metrics and soleus muscle injuries in professional football (soccer) players

**DOI:** 10.1002/jeo2.70755

**Published:** 2026-08-06

**Authors:** Luca Benedetti, Matthew Buckthorpe, Stefano Di Paolo, Francesco Della Villa, Gianni Nanni

**Affiliations:** ^1^ Medical and Performance Department Bologna Football Club Bologna Italy; ^2^ Education and Research Department, FIFA Medical Centre of Excellence Isokinetic Medical Group Bologna Italy; ^3^ FIFA Medical Centre of Excellence Isokinetic Medical Group London UK; ^4^ Faculty of Sport, Technology and Health Sciences St Mary's University Twickenham UK; ^5^ FIFA Medical Centre of Excellence Isokinetic Medical Group Bologna Italy

**Keywords:** calf, fatigue, injury epidemiology, injury prevention, muscle injury, training load

## Abstract

**Purpose:**

To investigate the association between external GPS workload metrics and soleus muscle injuries (SMI) in elite professional football.

**Methods:**

A retrospective cohort study was conducted across seven consecutive seasons (2017/2018–2023/2024) in a single Italian Serie A first‐team squad. One hundred and sixty player‐season observations were included (25.4 ± 3.2 years; goalkeepers excluded), with 4704 match‐hours and 31,686 training‐hours recorded. SMIs were defined as time‐loss soleus muscle complaints (excluding contact injuries) diagnosed by the club medical staff in accordance with FIFA consensus definitions and classified as structural or non‐structural. External loads were quantified using 50 Hz GPS (Stats Perform) and included total distance, high‐intensity distance (≥16 km·h^−1^), sprint distance (≥25 km·h^−1^), and acceleration/deceleration distances (≥±2.0 m·s^−2^). For injured players, workloads were averaged across the 7‐ and 28‐day window preceding injury (excluding injury day) and compared to control periods of equal duration. Between‐player comparisons contrasted injured players' injury periods with control periods in non‐injured players.

**Results:**

Twenty‐nine SMIs (52% were nonstructural) occurred in 17 players. Injured players were older than noninjured players (29.2 ± 3.9 vs. 25.0 ± 3.2 years; *p* = 0.041). Most injuries occurred in training (83%); incidence was 0.76/1000 training‐hours and 1.06/1000 match‐hours; injury burden was 10.86 days/1000 hours. No within‐player differences were observed between injury and control weeks for any workload metric (*p* > 0.05). Over 28 days, injured players demonstrated lower sprint distance versus their control month (−37%; *p* = 0.014; *d* = −0.71). For between‐player analyses, deceleration distance was higher in the 7‐day injury period versus noninjured controls (+17%; *p* = 0.041; *d* = 0.44), with no 28‐day differences.

**Conclusion:**

SMIs imposed a substantial burden in elite football and were not preceded by acute workload spikes relative to players' own control periods. Findings suggest SMIs may occur within a context of sustained high training volumes, with modest elevations in short‐term deceleration exposure and reduced longer‐term sprint distance observed prior to injury.

**Level of Evidence:**

Level III.

**Abbreviations**
a: alpha
ACC: accelerations
cm: centimetre
CMI: calf muscle injury
DEC: decelerations
ECIS: Elite Club Injury Study
FIFA: Fédération Internationale de Football Association
GPS: global positioning system
HID: high‐intensity distance
Hz: hertz
KG: kilograms
Km: kilometre
M: metre
SD: sprint distance
SMI: soleus muscle injury
TD: total distance

## INTRODUCTION

Muscle injuries remain a significant cause of time‐loss in professional football (soccer), with substantial consequences for team performance, player health and availability [27, 28, 39]. Muscle injuries account for around a third of all injuries [18, 19, 21] with a notable increase observed in relation to cumulative match exposure [4]. While most research has focused on hamstring muscle injuries, being the predominant muscle injury [21], calf muscle injuries (CMI) also represent a clinically important problem. CMIs account for ∼13% of all muscle injuries in elite football [18], with the majority of these involving the Soleus (∼70%) rather than the Gastrocnemius complex [30, 52]. These injuries often occur during critical periods of the season [40]. They are also associated with high recurrence rates [18, 52] and longer and more variable time‐loss versus other lower limb muscle injuries [18, 21]. Despite their frequency and impact, research on CMIs and specifically soleus muscle injury (SMIs) is limited.

Understanding injury aetiology is fundamental to both the prevention and management of injuries in sport [2, 53]. Musculoskeletal injury ultimately represents mechanical failure, occurring either when a single high‐magnitude load exceeds tissue capacity or when repetitive submaximal loading surpasses the tissue's fatigue tolerance [38]. Within this framework, appropriate management of training load is required to optimise fitness while minimising injury risk [3, 23, 57]. Both insufficient chronic loading and excessive loading, particularly when acute demands exceed a player's underlying fitness, have been associated with elevated injury risk in football and other team sports [6, 7, 37, 41, 42, 56]. A substantial body of literature has examined associations between training load and lower‐limb injury across team sports, including football. However, the effectiveness of workload monitoring as an injury prevention strategy remains debated, with inconsistent associations reported between workload metrics and injury occurrence [35]. A major limitation of this literature is that most studies aggregate all lower‐limb noncontact or soft‐tissue injuries into a single category when examining workload–injury relationships [6, 7, 37, 41, 42, 56]. Given the complex, multifactorial nature of sports injury [5], and the clear evidence that different injuries exhibit distinct biomechanical mechanisms and risk profiles [8, 9, 10, 11, 12, 13, 14, 24], such aggregation is likely to obscure clinically meaningful associations between specific workload metrics (e.g., running volume vs. sprint exposure) and specific injury pathologies.

The soleus muscle is particularly relevant in this context. As the primary contributor to propulsive force during running, it generates more mechanical work at sub‐maximal running velocities than any other lower‐limb muscle [15]. Unlike the knee flexors or hip extensors, soleus activation and force output remain consistently high across a wide range of running velocities [15]. The soleus muscle plays a critical role in ankle joint stability, postural control, braking, and regulation of leg stiffness during dynamic sporting tasks [15, 29, 46]. SMIs are disproportionately common versus gastrocnemius injuries in running‐based sports such as football [30, 52] and are frequently considered fatigue‐related injuries [18], likely reflecting sensitivity to cumulative running volume, particularly total distance (TD) and submaximal running load. This contrasts with other muscle groups in football and other sports, which tend to be more sensitive to high‐speed running or sprint exposure, such as hamstring injuries [47, 51]. Research exploring workload–injury relationships in CMI is extremely limited [52], with no study to date specifically examining the association between external workload and SMIs in football. An understanding of the soleus‐specific workload and injury relationship may enable more targeted injury prevention and rehabilitation and return‐to‐play strategies in football.

As such, the present study aimed to investigate the association between external training load metrics and the incidence of SMIs in professional football players. The hypothesis was formulated that an increased risk of SMIs would be associated with greater training loads, particularly when accumulated rapidly over short periods.

## METHODS

### Study design

This was a retrospective study conducted over seven consecutive seasons (2017/2018 to 2023/2024), involving the first team players of a single professional football club, competing in the Italian Serie A. Injury and training load data were collected as part of routine team monitoring procedures. SMIs were diagnosed and classified by the club's medical staff, while training and match loads were recorded via GPS technology.

### Participants

The study population comprised 160 player‐season observations of professional football players. Some players were included across multiple seasons if they remained in the squad; however, each player‐season was treated as an independent data point (age, 25.4 ± 3.2 years; range: 19–34 years). Player positions were: 64 defenders (40%), 56 midfielders (35%) and 40 forwards (25%). Goalkeepers were excluded. Total exposure during the study period was 4704 h for matches and 31,686 h for training. The following exclusion criteria were applied: (1) transfer to another club during the season; (2) absence of a first‐team contract and (3) presence of a medical complaint during the selected control periods.

Data were collected prospectively as part of routine medical and performance monitoring within the club across multiple seasons (2017/2018–2023/2024), and the present analyses were conducted retrospectively. Ethical approval was obtained from the Bioethical Committee of the University of Bologna (n° 0007503 of 08/01/2025), which specifically covered the retrospective use of anonymised routine monitoring data for research purposes. As part of their professional contractual agreements, players provided consent for the collection and use of anonymised medical and performance data for research and audit purposes in accordance with standard practice in elite professional sport.

### Injury and exposure data collection

The team's medical staff conducted a thorough diagnostic evaluation and recorded all injuries in accordance with the FIFA consensus statement for football injury studies [22]. A SMI was defined as any muscle‐related complaint involving the soleus muscle that resulted in time‐loss, defined as the player being unable to fully participate in at least one subsequent training session or match. The categorisation of injuries was based on the underlying cause, with acute injuries being attributed to a specific, identifiable event, and overuse injuries resulting from cumulative microtrauma without a discernible inciting incident, as defined by the consensus statement on injury definitions and data collection procedures in football [22]. These were categorised as either structural or non‐structural based on clinical assessment and imaging, in accordance with internal medical protocols [20]. Structural injuries were defined as those demonstrating macroscopic fibre disruption on MRI, whereas non‐structural injuries were defined as clinical soleus‐related time‐loss injuries without clear fibre disruption on imaging. MRI diagnoses were made by experienced sports medicine radiologists in collaboration with team physicians according to standard clinical practice. Formal inter‐rater reliability was not assessed. Direct contact injuries (e.g., contusions) were excluded from the analysis. Severity was defined by the number of days of absence: minimal (1–3 days), mild (4–7 days), moderate (8–28 days) and severe (>28 days).

The team's athletic trainers were responsible for recording exposure data, which included only on‐pitch training and match hours. Off‐pitch activities such as gym‐based strength sessions, recovery modalities, and preactivation exercises were not tracked using GPS and were therefore excluded from exposure time calculations. The incidence of injury was calculated as the number of SMIs per 1000 h of exposure. The functional impact was then quantified by calculating the number of days lost per 1000 h of exposure, thus providing a key indicator for evaluating the seasonal impact of soleus injuries on player availability and team management. Training exposure was defined as the total duration of on‐field training sessions, from the beginning of the warm‐up to the end of the final drill, excluding gym‐based or rehabilitation activities.

### Weekly external load monitoring

The external loads experienced by the athletes during training sessions and competitive matches were monitored via 50 Hz portable GPS units with integrated inertial sensors (K‐SPORT; Stats Perform). The validity and reliability of this K 50 model, which features sensor fusion between GPS and inertial technologies has been confirmed in a validation study [36], which reported a typical error of 1.9% and a coefficient of variation of 2.1% for distance measurement during dynamic movements. To minimise inter‐unit variability, each player consistently wore the same device across all sessions. Units were positioned between the scapulae at the level of the thoracic spine using a tight‐fitting vest to reduce movement artefacts. In accordance with manufacturer guidelines, devices were activated at least 15 min before the start of each training session and official match to ensure proper satellite lock and data reliability. Following the conclusion of the training practice or match, the GPS accelerometer data were downloaded to a personal computer and analysed using a customised software package (K‐Fitness; Stats‐Perform).

The physical performance variables selected for analysis included TD, high‐intensity distance [HID, defined as distance covered at speeds ≥16 km·h^−1^, including sprint distance (SD)], SD (defined as distance covered at speeds ≥ 25 km·h^−1^), as well as distances covered during moderate‐to‐high intensity accelerations (ACC, ≥2.0 m·s²) and decelerations (DEC, ≤–2.0 m·s²). GPS‐accelerometer data were also used to derive training and match durations (in minutes), which were summed to calculate total weekly on‐pitch exposure [52]. Only field‐based activities were included in this analysis. In cases of missing external load data (e.g., GPS dropout, device malfunction), individual values were estimated using the player's own recent data (e.g., same‐day session average or rolling weekly average), when available. If individual data were not available, team averages for the same position group and session type (training or match) were used as a substitute. This approach was applied consistently across both training sessions and official matches, and concerned fewer than 5% of the total data points. Similar strategies to handle GPS‐related missing data are commonly adopted in team‐sport monitoring research to minimize bias and preserve dataset validity [25, 43].

### Statistics

Descriptive statistics were presented as mean ± standard deviation for continuous variables, and as counts and percentages for categorical variables. Normality of continuous data was assessed using the Shapiro–Wilk test and Q–Q plots.

Workload metrics (TD, HID, SD, ACC and DEC) for the injured players were averaged across the injury week (the 7 days immediately preceding the injury, excluding the day of injury) and injury month (the 28 days immediately preceding the injury, excluding the day of injury).

These were compared to corresponding control periods of equal duration (7‐ and 28‐day windows). Control periods were selected from the same player within the same season where possible and were required to be free from injury, rehabilitation, and return‐to‐play phases. Periods were selected based on data completeness and availability from timeframes outside the injury window. Equivalent control periods were also extracted for noninjured players.

Paired‐samples *t*‐tests were used to compare injury and control periods within injured players, while independent‐samples *t*‐tests were used to compare injury periods in injured players with control periods in non‐injured players. Results were reported as mean difference and percentage change (Δ%). Cohen's *d* effect sizes were calculated and interpreted as trivial (<0.2), small (0.2–0.49), moderate (0.5–0.79), and large (≥0.8). All analyses were conducted using R (version 4.3.3), with statistical significance set at *p* < 0.05.

A power analysis was conducted using G*Power (version 3.1.9.7) based on a previous study with similar rationale [52]. The TD metric with a difference of 18% (21,586.60 ± 4100.48 m vs. 25,117.41 ± 4884.93 m) between injured and noninjured players, an effect size Cohen's *d* = 1.01, and an allocation ratio of 3:1 (noninjured:injured) were retrieved from the study [52]. To keep the sample size calculation more conservative, an allocation ration of 4:1 with an effect size = 0.6 were adopted. Considering *α* = 0.05, and power = 0.8, the total sample size was estimated in 140 players‐season observations.

## RESULTS

### Injury incidence and characteristics

Within the overall cohort, 29 SMIs were recorded across 17 players, corresponding to 18% of the 160 player‐season observations. Of these, nine players sustained a single injury, six sustained two injuries, and two players experienced four SMIs over the observation period. Injured players were significantly older (29.2 ± 3.9 years) than noninjured players (25.0 ± 3.2 years) (*p* = 0.04) (Table 1). Of the 29 injuries, 15 were classified as structural and 14 as nonstructural.

**Table 1:** Descriptive statistics of demographic, anthropometric and physiological variables in injured and noninjured players.

	All	Injured (*N* = 29)	Noninjured (*N* = 131)	*p*‐value
Age (years)	27.1 ± 3.6	29.2 ± 3.9	25.0 ± 3.2	**0.041**
Height (cm)	183.9 ± 6.4	183.3 ± 5.9	184.4 ± 6.9	0.565
Body mass (kg)	83.5 ± 7.2	82.5 ± 6.8	84.5 ± 7.5	0.632
Position				
Defender	59	9 (15%)	50 (85%)	
Midfielder	57	9 (16%)	48 (84%)	
Forward	44	11 (25%)	33 (75%)	0.244
Overall	160			

*Note*: Values are presented as mean ± standard deviation for continuous variables and as number (percentage) for categorical variables. *p* < 0.05 indicates statistical significance.

The distribution of injuries by position was as follows: defenders accounted for 31% of injuries, midfielders 31% and forwards 37%. When considering the proportion of players injured within each position, approximately 1 in 4 forwards sustained an injury, compared with fewer than 1 in 6 defenders and midfielders.

Of the injuries sustained, 24 (82.8%) occurred during training and 5 (17.2%) during matches. The overall incidence was 0.76 injuries per 1000 h of training exposure and 1.06 injuries per 1000 match hours. The total injury burden, measured in terms of the number of days of absence per 1000 h of exposure, was 10.86 days per 1000 h. The mean time of absence per injury was 12.9 ± 13.3 days.

### Injury versus control period workload in injured players

During the 7‐days period, no differences in workload metrics were found between injury and control period for the injured players (*p* > 0.05, Table 2). During the 28‐days period, players sustaining a SMI showed lower SD in relation to their own control period (Δ = 37%, *p* = 0.014, Table 3).

**Table 2:** Comparison of the workload metrics of players sustaining a soleus muscle injury between the injury week and a control week and between injured and noninjured players (7‐day period).

7‐days	Injured players		Noninjured players	Injured: injury vs. control week			Injured vs. noninjured players		
	Injury week	Control week	Control week	Δ%	*p*‐value	ES	Δ%	*p*‐value	ES
TD	35,245 ± 8124	36,973 ± 9069	32,496 ± 7767	−5%	0.485	−0.20	8%	0.098	0.35
HID	5652 ± 1964	6275 ± 2094	5081 ± 1639	−11%	0.251	−0.31	10%	0.122	0.32
SD	261 ± 199	357 ± 233	223 ± 207	−37%	0.110	−0.45	14%	0.397	0.18
ACC	1841 ± 691	1758 ± 930	1592 ± 701	5%	0.576	0.10	14%	0.105	0.36
DEC	1657 ± 663	1593 ± 774	1375 ± 613	4%	0.599	0.09	**17%**	**0.041**	**0.44**

*Note*: values are presented as mean ± standard deviation; bold *p*‐values indicate statistical significance.

Abbreviations: ACC, acceleration distance (≥2.0 m·s²); DEC, deceleration distance (≥2.0 m·s²), ES, Cohen's *d* effect size; HID, high intensity distance (distance covered in metres at speeds ≥16 km·h^−1^, including sprint distance); SD, sprint distance (distance in metres covered at speeds ≥25 km·h^−1^); TD, total distance (m). Bold values indicate significant findings.

**Table 3:** Comparison of the workload metrics of players sustaining a soleus muscle injury between the injury month and a control month and between injured and noninjured players (28‐day period).

28‐days	Injured players		Noninjured players	Injured: injury vs. control month			Injured vs. noninjured players		
	Injury month	Control month	Control month	Δ%	*p*‐value	ES	Δ%	*p*‐value	ES
TD	136,168 ± 29,622	147,377 ± 20,943	133,789 ± 26,023	−8%	0.080	−0.44	2%	0.685	0.09
HID	21,457 ± 5486	24,562 ± 4959	21,959 ± 6737	−14%	0.100	−0.59	−2%	0.724	−0.08
SD	1009 ± 600	1381 ± 443	1105 ± 800	**−37%**	**0.014**	**−0.71**	−10%	0.558	−0.14
ACC	7043 ± 2526	7105 ± 3046	6465 ± 2775	−1%	0.851	−0.02	8%	0.346	0.22
DEC	6328 ± 2416	6574 ± 2728	5557 ± 2392	−4%	0.932	−0.10	12%	0.157	0.32

*Note*: values are presented as mean ± standard deviation; bold *p*‐values indicate statistical significance.

Abbreviations: ACC, acceleration distance (≥2.0 m·s²); DEC, deceleration distance (≥2.0 m·s²); ES, Cohen's *d* effect size; HID, high intensity distance (distance covered in metres at speeds ≥16 km·h^−1^, including sprint distance), SD, sprint distance (distance in metres covered at speeds ≥25 km·h^−1^), TD, total distance (m). Bold values indicate significant findings.

### Injured versus noninjured players workload

During the 7‐days period prior to injury, players reported higher DEC distance than noninjured players (Δ = 17%, *p* = 0.041, Table 2). No differences were noted during the 28‐days period (*p* > 0.05).

## DISCUSSION

This is the first study to specifically examine the association of external workload metrics during training and matches and SMIs in elite male football. The main findings were that SMIs imposed a substantial injury burden on the squad, with an average of approximately four injuries per season. Injuries were more common in training than matches and were more common in older players and those with a history of SMIs. In the week preceding injury, injured players displayed similar total workloads to their own control week, although DEC workloads were moderately higher, with trends for greater TD, ACC and HID compared to non‐injured players. Over the 4‐week period prior to injury, workload exposure was largely comparable to control periods, except for lower SD relative to players own 4‐week control period.

### Epidemiology

The incidence and burden of SMIs observed in the present cohort were markedly higher than those reported in the UEFA Elite Club Injury Study (ECIS) for CMIs [18, 26], although they were comparable to more recent data from Spanish professional football [52]. Across the seven‐season observation period, 29 SMIs were recorded, equating to ∼4 SMIs per season, and an injury burden of 10.86 days per 1000 h. In contrast, Ekstrand et al. [18] reported that elite European teams typical sustain approximately two CMIs per season, with a burden of 4.6 days per 1000 h. This represents a more than two‐fold greater injury burden in the present cohort, despite our analysis being restricted specifically to SMIs, rather than all CMIs.

The majority of SMIs occurred during training (83%), with few (17%) sustained during matches. This corresponds to only a moderately higher risk (∼40%) in matches versus training (0.74 vs. 1.06 injuries per 1000 h). This contrasts with previous reports on CMIs, which suggests a five‐to‐six‐fold greater match risk versus training, with ∼70%–75% of injuries occurring during matches [18, 52]. This discrepancy may reflect the present study's exclusive focus on SMIs, in contrast to broader classification of CMIs in prior surveillance studies. Recent work has demonstrated that SMIs account for around 70% of calf injuries in professional football [30, 52]. The higher proportion of training‐related SMIs observed in the present cohort therefore supports the premise that SMIs pathology may be more closely linked to cumulative training exposure than acute match demands. This supports research showing that CMIs in elite football (ECIS) are not impacted by congested match play, like other lower limb muscle injuries [4].

More than half of all injuries were classified as non‐structural with relatively short time‐loss, suggesting that functional SMIs may have been under‐reported in earlier surveillance studies. The mean absence per injury (12.9 days) was slightly lower than that reported previously for CMIs (∼15 days) [18, 52]. Improvements in diagnostic awareness of soleus‐specific pathology likely contribute to the higher incidence observed.

Injured players were significantly older than non‐injured players, consistent with previous research [18, 26, 52]. No differences were observed for height, body mass, or playing position. Re‐injury was common, with nearly half of injured players sustaining multiple SMIs. Prior injury is a well‐established risk factor for calf and soleus injuries [24], likely reflecting persistent deficits in tissue capacity, neuromuscular control, and load tolerance.

### Workload and SMIs

This is the first study to specifically examine the relationship between external workload and SMIs, in elite football, rather than grouping these injuries under CMIs or lower limb muscle categories. A recent pilot study in Spanish professional football examining CMIs reported an acute increase in workload (16%–37%) during the 7‐day period preceding injury [52]. In contrast, the present pathology‐specific analysis of SMIs did not demonstrate comparable acute workload elevations in the week preceding injury, with workload values largely similar to players' own control periods. Differences in injury classification (CMIs vs. SMIs), cohort characteristics, and analytic approach (within‐player comparisons in the present study versus aggregated injury analyses in prior work) may partly explain these contrasting findings. These observations suggest that workload patterns associated with soleus‐specific pathology may differ from those reported for broader calf muscle injury groupings, although direct comparisons between studies should be interpreted cautiously. As such, injured players did not experience an acute increase in workload immediately prior to injury. This contrasts with several studies on lower‐limb soft‐tissue injury that have linked acute workload spikes and elevated acute: chronic workload ratios to increased injury risk in football [6, 7, 17, 37] and other team sports [16, 31, 32, 33, 42], suggesting that the mechanisms underpinning SMIs may differ from those proposed for other muscle injuries in football.

Similarly, minimal change and in some cases a slight reduction was observed in the 4‐week workload preceding injury compared with players' control 4‐week period. To our knowledge this is the first study to document 4‐week workload exposure prior to CMIs/SMIs specifically. Previous work examining longer‐term loading patterns for aggregated lower‐limb injuries has generally reported both increased [6, 7] and decreased injury risk with high 3‐to‐4‐week training loads [17] highlighting the complexity of more chronic workload‐injury relationships. Together, these findings indicate that SMIs in this cohort were not preceded by acute (7‐day) or more sustained (4‐week) elevations in external workload metrics. Bowen et al. [7] reported that a low amount of TD accumulated over 4‐weeks increased the risk of sustaining non‐contact injury, particularly when low chronic loading was followed by an acute spike prior to injury.

Between player comparison further supported the observation that changes in workload were not associated with injury. Injured players demonstrated only modestly higher 7‐day DEC workloads than un‐injured players (+17%, ES, 0.40) with similarly small‐to‐moderate effect sizes for TD, ACC and HID. This is the first study to contrast workload exposure specifically between players sustaining SMI and un‐injured players. Most previous investigations have pooled all lower‐limb soft‐tissue or non‐contact injuries into a single category, limiting the ability to draw pathology‐specific inferences.

While acute workload change was minimal, the absolute workload values in this cohort, specifically TD were notably high when compared with those reported previously. Seven‐day TD values in the present cohort (35–37 km) were approximately 40%–70% greater than those reported in the Spanish cohort studied by Soler et al. [52]. Based on published classifications, where <24 km is considered low, 24–30 km low‐to‐moderate, >30–31 km high and >37 km very high [7, 37], all 7‐day TD workloads in the present study would be classified as high‐to‐very‐high. A similar pattern was evident for 4‐week workloads, where both injured and uninjured players accumulated very high chronic TD exposures (>134 km across 28 days). Therefore, despite the absence of acute workload spikes, players in this cohort were consistently exposed to very high cumulative TD/running volumes. Four‐week loads are typically conceptualised as markers of chronic loading and physical preparedness (i.e., “fitness”) and are often considered protective against injury when appropriately progressed [23]. It is therefore plausible that in some players these loads were protective, whereas in others, particularly those with prior injury or age‐related reductions in tissue capacity, they may have exceeded individual tissue load‐tolerance capacity. Collectively, these contrasting findings suggest that different workload patterns may be associated with CMIs versus soleus‐specific injuries, although direct comparisons between studies should be made cautiously.

These findings have important mechanistic implications for SMIs. Jogging and submaximal running impose substantial repetitive loads on the plantar‐flexor muscle–tendon complex, with the soleus acting as the primary contributor to propulsive force during these activities [15]. Unlike the hamstrings or hip extensors, which experience their greatest mechanical demands at high running speeds, soleus activation remains high across a wide range of running velocities, including low‐ and moderate‐speed running [15]. During submaximal running, athletes typically experience vertical ground reaction forces of approximately 2–3 times body mass per step [15, 45, 48] with a large proportion of this force generated by the ankle plantar flexors [15]. At faster speeds, soleus force contribution decreases due to reduced ground contact time and increased reliance on proximal musculature [15]. Consequently, TD may reflect a proxy of cumulative plantar‐flexor exposure rather than peak mechanical demand. From this perspective, the consistently high TD exposure observed in the present cohort may represent a contributing mechanical context within which SMIs occurred in this cohort.

The biomechanical specificity of soleus loading also helps explain the discrepancy between the present findings and the sprint‐dominated workload–injury relationships commonly reported for hamstring injuries [47, 51]. Whereas hamstring strain risk is strongly linked to high‐speed running and sprint exposure, SMIs appear to be more sensitive to prolonged submaximal loading and fatigue accumulation. In this context, the significantly lower 4‐week SD observed prior to injury could reflect reduced exposure to high‐velocity loading, potentially leading to a loss of load variability and a greater relative contribution of submaximal running to overall mechanical stress.

Finally, the fact that many SMIs occurred in older players and as re‐injuries suggests that workload effects are likely moderated by intrinsic risk factors such as age‐related reductions in tissue capacity and residual deficits following prior injury. Previous injury is a well‐established risk factor for CMIs and SMIs [24, 26], and it is plausible that given the same absolute workload exposure, previously injured or older players may operate closer to their individual tissue tolerance limits. Although the present study was not sufficiently powered to stratify workload–injury relationships by injury history, future research should examine how workload interacts with intrinsic risk modifiers such as age, prior SMI, plantar‐flexor strength, and ankle dorsiflexion range of motion, to name a few.

#### Methodological considerations

The present study has several important methodological strengths and limitations that should be considered when interpreting the findings. Key strengths include the seven consecutive seasons of data collection within a consistent elite professional football environment, the homogeneous cohort of first‐team players, and the prospective recording of both injury and external workload data as part of routine club monitoring. Workload associations were examined using both within‐player (injury vs. control periods) and between‐player (injured vs. noninjured players) comparisons across defined 7‐ and 28‐day time windows. The extended observation period enabled a pathology‐specific investigation focused exclusively on SMIs, rather than grouping all calf or lower limb muscle injuries together, thereby improving biomechanical and clinical specificity.

Despite these strengths, several methodological considerations warrant careful interpretation. Although injury and workload data were prospectively collected, the present study employed a retrospective analytical design. Accordingly, the findings should be interpreted as exploratory associations rather than evidence of causality. The relatively small number of SMIs available for analysis (*n* = 29) limits statistical power and restricts the ability to detect small‐to‐moderate exposure‐injury relationships [1, 2, 3]. While comparable to many single‐club longitudinal workload studies in elite football [7, 37, 42, 52], substantially larger datasets are required to examine interaction effects and multifactorial injury models [38, 50]. The limited number of injuries also precluded multivariate modelling and analysis was therefore restricted to univariable comparisons. Contemporary theoretical models conceptualise sports injury as the result of dynamic, non‐linear interactions between multiple intrinsic and extrinsic risk factors, including age, strength, fatigue, movement strategy, and workload [2, 5, 57]. Future research should therefore examine workload–SMI relationships within larger cohorts that allow for multivariable and interaction‐based modelling.

Potential sources of bias should also be acknowledged. Selection bias may arise from the single‐club cohort design, and survivorship bias may have occurred across seasons due to player transfers, differences in squad status, or variable exposure time. Information bias is possible given the reliance on GPS‐derived workload metrics as external proxies of mechanical demand. Although injury diagnoses were made by experienced medical staff in accordance with consensus definitions, misclassification cannot be entirely excluded. In addition, injured players were significantly older than noninjured players, and prior injury history was not formally adjusted for in the analyses. Residual confounding by age, prior injury, or other intrinsic factors (e.g., strength or neuromuscular capacity) is therefore possible and limits causal interpretation.

The unit of analysis also warrants clarification. Some players contributed data across multiple seasons, and a small number sustained more than one SMI. Analyses treated player‐season observations as independent and clustering within players was not formally modelled. This approach may underestimate variance and overstate statistical precision. Given the modest number of injuries, more complex mixed‐effects or cluster‐adjusted models were not considered feasible without risking model instability; results should therefore be interpreted with appropriate caution.

Missing GPS data (<5%) were imputed using recent individual values or, when unavailable, team or positional averages. While this approach preserved sample size and analytic stability, it may attenuate variance and influence effect estimates. Sensitivity analyses were not performed due to the limited number of events, and the potential impact of imputation should be considered when interpreting the findings.

Finally, multiple workload variables were examined across two time windows using both within‐ and between‐player comparisons. No formal adjustment for multiple comparisons was applied. Accordingly, the analyses should be interpreted as exploratory, and statistically significant findings should be considered in light of potential type I error inflation.

To enhance analytic stability and reduce collinearity between workload metrics, primary variables were limited to TD, HID, SD and moderate‐to‐high intensity acceleration and deceleration (≥±2.0 m·s⁻²), capturing key dimensions of volume, intensity, and change‐of‐velocity demands. It is possible that using higher thresholds for ACC and DEC may have revealed different associations with SMIs [44, 49, 55]. Although GPS technology provides practical estimates of external workload in elite football, it cannot directly quantify internal tissue loading, joint moments, muscle–tendon forces, or fatigue state [34, 54]. Workload metrics should therefore be interpreted as external proxies of mechanical demand rather than direct measures of soleus muscle loading.

Future research integrating larger multi‐club cohorts, multivariable modelling, biomechanical assessment, and neuromuscular profiling would further advance understanding of SMI mechanisms and workload interactions.

#### Practical implications

SMIs appear to represent a meaningful injury burden in elite football, greater than previously recognised, with the present cohort sustaining ~4 SMIs per season and exhibiting a substantially greater injury burden than typically reported in the literature. Most injuries were classified as minor to moderate, with a high proportion of nonstructural (functional) injuries. This pattern is consistent with a fatigue‐related aetiology and likely reflects the exceptionally high chronic TD workloads observed in this cohort.

Given the unique functional role of the soleus as the primary contributor to propulsion during jogging and running, TD and submaximal running volume may represent relevant indicators of cumulative plantar‐flexor exposure in football, acknowledging that GPS‐derived metrics provide indirect estimates of internal tissue loading. From a practical perspective, these findings indicate that monitoring and regulating chronic TD and/or running loads rather than focusing solely on sprint and change of velocity (ACC, DEC) exposure should form a central component of SMI risk management strategies. Sustained exposure to high running volumes, even in the absence of acute workload spikes, is likely to exert a meaningful and cumulative injury risk for SMIs in elite football players.

## CONCLUSION

SMIs exert a meaningful injury burden in elite football, with this cohort sustaining approximately four SMIs per season. Injuries were not associated with acute increases in training load prior to injury but occurred in the context of consistently high chronic training volumes, particularly TD and running loads. These findings suggest that sustained high running volumes may characterise the workload context in which SMIs occurred in this cohort and warrant consideration within monitoring strategies in elite football.

## AUTHOR CONTRIBUTIONS

Gianni Nanni and Luca Benedetti conceived the idea for the study and oversaw the longitudinal data collection and/or data management. Gianni Nanni oversaw the medical team prised with injury diagnosis. Luca Benedetti oversaw collection of GPS workload and training exposure data. Luca Benedetti, Matthew Buckthorpe and Stefano Di Paolo performed the data analysis, interpreted results and wrote the manuscript, with all other authors providing intellectual input on draughts versions of the manuscript. All authors approved the final version of the manuscript.

## CONFLICT OF INTEREST STATEMENT

The authors declare no conflicts of interest.

## FUNDING INFORMATION

The authors have no funding to report.

## ETHICS STATEMENT

The study received ethical approval from the Bioethical Committee of the University of Bologna (n° 0007503 of 08/01/2025).

## Data Availability

The data that support the findings of this study are not publicly available due to privacy and contractual restrictions with the participating club(s), but may be available from the corresponding author upon reasonable request and with permission of the participating organisation.

## References

[1] Bahr R , Holme I . Risk factors for sports injuries—a methodological approach. Br J Sports Med. 2003;37(5):384–392. 14514527 10.1136/bjsm.37.5.384 PMC1751357

[2] Bahr R , Krosshaug T . Understanding injury mechanisms: a key component of preventing injuries in sport. Br J Sports Med. 2005;39(6):324–329. 15911600 10.1136/bjsm.2005.018341 PMC1725226

[3] Banister EW , Calvert TW , Savage MV , Bach T . A systems model of training for athletic performance. Aust J Sports Med. 1975;7:57–61.

[4] Bengtsson H , Ekstrand J , Hägglund M . Muscle injury rates in professional football increase with fixture congestion: an 11‐year follow‐up of the UEFA Champions League injury study. Br J Sports Med. 2013;47(12):743–747. 23851296 10.1136/bjsports-2013-092383

[5] Bittencourt NFN , Meeuwisse WH , Mendonça LD , Nettel‐Aguirre A , Ocarino JM , Fonseca ST . Complex systems approach for sports injuries: moving from risk factor identification to injury pattern recognition‐narrative review and new concept. Br J Sports Med. 2016;50(21):1309–1314. 27445362 10.1136/bjsports-2015-095850

[6] Bowen L , Gross AS , Gimpel M , Li FX . Accumulated workloads and the acute: chronic workload ratio relate to injury risk in elite youth football players. Br J Sports Med. 2017;51(5):452–459. 27450360 10.1136/bjsports-2015-095820 PMC5460663

[7] Bowen L , Gross AS , Gimpel M , Bruce‐Low S , Li FX . Spikes in acute:chronic workload ratio (ACWR) associated with a 5‐7 times greater injury rate in English Premier League football players: a comprehensive 3‐year study. Br J Sports Med. 2020;54(12):731–738. 30792258 10.1136/bjsports-2018-099422 PMC7285788

[8] Buckthorpe M , Pirli Capitani L , Olivares‐Jabalera J , Olmo J , Della Villa F . Systematic video analysis of ACL injuries in professional Spanish male football (soccer): injury mechanisms, situational patterns, biomechanics and neurocognitive errors—a study on 115 consecutive cases. BMJ Open Sport & Exercise Medicine. 2024;10(3):e002149. 10.1136/bmjsem-2024-002149 PMC11440205 39351123

[9] Buckthorpe M , Pisoni D , Tosarelli F , Danelon F , Grassi A , Della Villa F . Three main mechanisms characterize medial collateral ligament injuries in professional male soccer‐blow to the knee, contact to the leg or foot, and sliding: video analysis of 37 consecutive injuries. J Orthop Sports Phys Ther. 2021;51(12):611–618. 34784244 10.2519/jospt.2021.10529

[10] Buckthorpe M , Verhagen E , D'Hooghe P , Osti L , Di Paolo S , Della Villa F . Systematic video analysis of ankle sprain injuries in elite male football (soccer): injury mechanisms, situational patterns, biomechanics and neurocognitive errors study: a study on 140 consecutive players. Knee Surg Sports Traumatol Arthrosc. 2025;33(11):4035–4049. 40923438 10.1002/ksa.70049 PMC12582226

[11] Della Villa F , Buckthorpe M , Grassi A , Nabiuzzi A , Tosarelli F , Zaffagnini S , et al. Systematic video analysis of ACL injuries in professional male football (soccer): injury mechanisms, situational patterns and biomechanics study on 134 consecutive cases. Br J Sports Med. 2020;54:1423–1432. 32561515 10.1136/bjsports-2019-101247

[12] Della Villa F , Buckthorpe M , Tosarelli F , Zago M , Zaffagnini S , Grassi A . Video analysis of Achilles tendon rupture in male professional football (soccer) players: injury mechanisms, patterns and biomechanics. BMJ Open Sport Exerc Med. 2022;8(3):e001419. 10.1136/bmjsem-2022-001419 PMC9511658 36172398

[13] Della Villa F , Massa B , Bortolami A , Nanni G , Olmo J , Buckthorpe M . Injury mechanisms and situational patterns of severe lower limb muscle injuries in male professional football (soccer) players: a systematic video analysis study on 103 cases. Br J Sports Med. 2023;57(24):1550–1558. 37898508 10.1136/bjsports-2023-106850

[14] Della Villa F , Stride M , Bortolami A , Williams A , Davison M , Buckthorpe M . Systematic video analysis of ACL injuries in male professional English soccer players: a study of 124 cases. Orthop J Sports Med. 2025;13(2):23259671251314642. 39991648 10.1177/23259671251314642 PMC11843699

[15] Dorn TW , Schache AG , Pandy MG . Muscular strategy shift in human running: dependence of running speed on hip and ankle muscle performance. J Exp Biol. 2012;215(11):1944–1956. 22573774 10.1242/jeb.064527

[16] Duhig S , Shield AJ , Opar D , Gabbett TJ , Ferguson C , Williams M. Effect of high‐speed running on hamstring strain injury risk. Br J Sports Med. 2016;50(24):1536–1540. 27288515 10.1136/bjsports-2015-095679

[17] Ehrmann FE , Duncan CS , Sindhusake D , Franzsen WN , Greene DA . GPS and injury prevention in professional soccer. J Strength Cond Res. 2016;30(2):360–367. 26200191 10.1519/JSC.0000000000001093

[18] Ekstrand J , Hägglund M , Waldén M . Epidemiology of muscle injuries in professional football (soccer). Am J Sports Med. 2011;39(6):1226–1232. 21335353 10.1177/0363546510395879

[19] Ekstrand J , Hägglund M , Waldén M . Injury incidence and injury patterns in professional football: the UEFA injury study. Br J Sports Med. 2011;45(7):553–558. 19553225 10.1136/bjsm.2009.060582

[20] Leeder J , Gissane C , van Someren K , Gregson W , Howatson G. Cold water immersion and recovery from strenuous exercise: a meta‐analysis. Br J Sports Med. 2012;46(4):233–240. 21947816 10.1136/bjsports-2011-090061

[21] Ekstrand J , Krutsch W , Spreco A , van Zoest W , Roberts C , Meyer T , et al. Time before return to play for the most common injuries in professional football: a 16‐year follow‐up of the UEFA Elite Club Injury Study. Br J Sports Med. 2020;54(7):421–426. 31182429 10.1136/bjsports-2019-100666 PMC7146935

[22] Fuller CW , Ekstrand J , Junge A , Andersen TE , Bahr R , Dvorak J , et al. Consensus statement on injury definitions and data collection procedures in studies of football (soccer) injuries. Br J Sports Med. 2006;40(3):193–201. 16505073 10.1136/bjsm.2005.025270 PMC2491990

[23] Gabbett TJ . The training‐injury prevention paradox: should athletes be training smarter and harder? Br J Sports Med. 2016;50(5):273–280. 26758673 10.1136/bjsports-2015-095788 PMC4789704

[24] Green B , Pizzari T . Calf muscle strain injuries in sport: a systematic review of risk factors for injury. Br J Sports Med. 2017;51(16):1189–1194. 28259848 10.1136/bjsports-2016-097177

[25] Griffin J , Newans T , Horan S , Keogh J , Andreatta M , Minahan C . Acceleration and high‐speed running profiles of women's international and domestic football matches. Front Sports Act Living. 2021;3:604605. 33842879 10.3389/fspor.2021.604605 PMC8027246

[26] Hägglund M , Waldén M , Ekstrand J . Risk factors for lower extremity muscle injury in professional soccer: the UEFA Injury Study. Am J Sports Med. 2013;41(2):327–335. 23263293 10.1177/0363546512470634

[27] Hägglund M , Waldén M , Magnusson H , Kristenson K , Bengtsson H , Ekstrand J . Injuries affect team performance negatively in professional football: an 11‐year follow‐up of the UEFA Champions League injury study. Br J Sports Med. 2013;47:738–742. 23645832 10.1136/bjsports-2013-092215

[28] Hickey J , Shield AJ , Williams MD , Opar DA . The financial cost of hamstring strain injuries in the Australian Football League. Br J Sports Med. 2014;48:729–730. 24124035 10.1136/bjsports-2013-092884

[29] Hirata RP , Akagi R . Contributions of the soleus muscle to postural control and ankle stiffness during quiet standing. J App Physiol. 2022;132(2):353–360.

[30] Huber PJ , Schönnagel B , Dalos D , Frosch KH , Adam G , Welsch GH . Muscle injuries in 90 professional football players over 10 consecutive seasons: a comparison of two classification systems and their association with return‐to‐play time. Scand J Med Sci Sports. 2025;35(10):e70147. 41103097 10.1111/sms.70147 PMC12531594

[31] Hulin BT , Gabbett TJ , Blanch P , Chapman P , Bailey D , Orchard JW. Spikes in acute workload are associated with increased injury risk in elite cricket fast bowlers. Br J Sports Med. 2014;48:708–712. 23962877 10.1136/bjsports-2013-092524

[32] Hulin BT , Gabbett TJ , Lawson DW , Caputi P , Sampson JA. The acute:chronic workload ratio predicts injury: high chronic workload may decrease injury risk in elite rugby league players. Br J Sports Med. 2016;50:231–236. 26511006 10.1136/bjsports-2015-094817

[33] Hulin BT , Gabbett TJ , Caputi P , Lawson DW , Sampson JA. Low chronic workload and the acute:chronic workload ratio are more predictive of injury than between‐match recovery time: a two‐season prospective cohort study in elite rugby league players. Br J Sports Med. 2016;50:1008–1012. 26851288 10.1136/bjsports-2015-095364

[34] Impellizzeri FM , Marcora SM , Coutts AJ . Internal and external training load: 15 years on. Int J Sports Physiol Perform. 2019;14(2):270–273. 30614348 10.1123/ijspp.2018-0935

[35] Impellizzeri FM , Menaspà P , Coutts AJ , Kalkhoven J , Menaspà MJ . Training load and its role in injury prevention, part I: back to the future. J Athl Train. 2020;55(9):885–892. 32991701 10.4085/1062-6050-500-19 PMC7534945

[36] Izzo R , Angelucci S , Di Michele R . Validation of a portable GPS‐inertial measurement system for dynamic sport movements. Meas Phys Educ Exerc Sci. 2022;26(3):230–241.

[37] Jaspers A , Kuyvenhoven JP , Staes F , Frencken WGP , Helsen WF , Brink MS . Examination of the external and internal load indicators' association with overuse injuries in professional soccer players. J Sci Med Sport. 2018;21(6):579–585. 29079295 10.1016/j.jsams.2017.10.005

[38] Kalkhoven JT , Watsford ML , Coutts AJ , Edwards WB , Impellizzeri FM . Training load and injury: causal pathways and future directions. Sports Med. 2021;51(6):1137–1150. 33400216 10.1007/s40279-020-01413-6

[39] López‐Valenciano A , Ruiz‐Pérez I , Garcia‐Gómez A , Vera‐Garcia FJ , De Ste Croix M , Myer GD , et al. Epidemiology of injuries in professional football: a systematic review and meta analysis. Br J Sports Med. 2020;54:711–718. 31171515 10.1136/bjsports-2018-099577 PMC9929604

[40] Mallo J , Dellal A. Injury risk in professional football players with special reference to the playing position and training periodization. J Sports Med Phys Fitness. 2012;52(6):631–638. 23187326

[41] Malone S , Roe M , Doran DA , Gabbett TJ , Collins K . High chronic training loads and exposure to bouts of maximal velocity running reduce injury risk in elite Gaelic football. J Sci Med Sport. 2017;20(3):250–254. 27554923 10.1016/j.jsams.2016.08.005

[42] Malone S , Roe M , Doran DA , Gabbett TJ , Collins KD . Aerobic fitness and playing experience protect against spikes in workload: the role of the acute: chronic workload ratio on injury risk in elite Gaelic football. Int J Sports Physiol Perform. 2016;24:1–25. 10.1123/ijspp.2016-0090 27400233

[43] Malone S , Owen A , Mendes B , Hughes B , Collins K , Gabbett TJ . High‐speed running and sprinting as an injury risk factor in soccer: can well‐developed physical qualities reduce the risk? J Sci Med Sport. 2018;21(3):257–262. 28595870 10.1016/j.jsams.2017.05.016

[44] Maniar N , Schache AG , Cole MH , Opar DA . Lower‐limb muscle function during sidestep cutting. Journal of Biomechanics. 2019;82:186–192. 30448192 10.1016/j.jbiomech.2018.10.021

[45] Munro CF , Miller DI , Fuglevand AJ . Ground reaction forces in running: a reexamination. J Biomech. 1987;20(2):147–155. 3571295 10.1016/0021-9290(87)90306-x

[46] Neptune RR , Sasaki K , Kautz SA . The effect of walking speed on muscle function and mechanical energetics. Gait Posture. 2008;28(1):135–143. 18158246 10.1016/j.gaitpost.2007.11.004 PMC2409271

[47] Nilsson T , Fransson D , Borjesson M , Lundblad M , Ivarsson A . Hamstring injury risk in male professional football: do external training loads play a role? BMJ Open Sport Exerc Med. 2025;11(3):e002649. 10.1136/bmjsem-2025-002649 PMC12421611 40937318

[48] Nilsson J , Thorstensson A . Ground reaction forces at different speeds of human walking and running. Acta Physiol Scand. 1989;136(2):217–227. 2782094 10.1111/j.1748-1716.1989.tb08655.x

[49] Pandy MG , Lai AKM , Schache AG , Lin YC . How muscles maximize performance in accelerated sprinting. Scand J Med Sci Sports. 2021;31(10):1882–1896. 34270824 10.1111/sms.14021

[50] Riley RD , Snell KIE , Ensor J , et al. Minimum sample size for developing a multivariable prediction model: part I—continuous outcomes. Stat Med. 2019;38(7):1262–1275. 30347470 10.1002/sim.7993

[51] Ruddy JD , Pollard CW , Timmins RG , Williams MD , Shield AJ , Opar DA . Running exposure is associated with the risk of hamstring strain injury in elite Australian footballers. Br J Sports Med. 2018;52(14):919–928. 27884865 10.1136/bjsports-2016-096777

[52] Soler A , Agulló F , Hernández‐Davó J , Raya‐González J , Del Coso J , González‐Ródenas J , et al. Influence of the external workload on calf muscle strain injuries in professional football players: a pilot study. Sports Health. 2025;17(1):175–182. 38708680 10.1177/19417381241247754 PMC11569535

[53] van Mechelen W , Hlobil H , Kemper HCG . Incidence, severity, aetiology and prevention of sports injuries: a review of concepts. Sports Med. 1992;14:82–99. 1509229 10.2165/00007256-199214020-00002

[54] Vanrenterghem J , Nedergaard NJ , Robinson MA , Drust B . Training load monitoring in team sports: a novel framework separating physiological and biomechanical load‐adaptation pathways. Sports Med. 2017;47(11):2135–2142. 28283992 10.1007/s40279-017-0714-2

[55] Verheul J , Harper D , Robinson MA . Forces experienced at different levels of the musculoskeletal system during horizontal decelerations. J Sports Sci. 2024;42(23):2242–2253. 39545586 10.1080/02640414.2024.2428086

[56] Verstappen S , van Rijn RM , Cost R , Stubbe JH . The association between training load and injury risk in elite youth soccer players: a systematic review and best evidence synthesis. Sports Med Open. 2021;7(1):6. 33428001 10.1186/s40798-020-00296-1 PMC7801562

[57] Windt J , Gabbett TJ . How do training and competition workloads relate to injury? The workload‐injury aetiology model. Br J Sports Med. 2017;51(5):428–435. 27418321 10.1136/bjsports-2016-096040

